# Act Tough and Soft: Video Monitoring, Hongbao Gifts, and the Job Satisfaction of Domestic Workers

**DOI:** 10.3389/fpubh.2022.862162

**Published:** 2022-03-25

**Authors:** Anuo Yang, Shuangle Fu, Linping Liu, Changyu Fan, Maitixirepu Jilili

**Affiliations:** ^1^School of Social and Behavioral Sciences, Nanjing University, Nanjing, China; ^2^School of Public Administration, Hangzhou Normal University, Hangzhou, China; ^3^School of Sociology, Central China Normal University, Wuhan, China

**Keywords:** video monitoring, hongbao gifts, job satisfaction, perceived discrimination, domestic workers, labor control, mediating effect

## Abstract

There is a rapidly growing demand for domestic services among urban families in China. However, domestic work remains a low-status occupation with a high turnover rate. Focusing on the job satisfaction of domestic workers is useful to interpret this phenomenon. We investigate how the job satisfaction of domestic workers in China is affected by to two distinct labor control strategies used by their employers: the installation of video-monitoring devices in employers' homes (a “tough” control strategy), and the Chinese custom of giving monetary gifts, or “hongbao” (a “soft” control strategy). By analyzing data from surveys of domestic workers in four cities in China (*N* = 699), we find that video monitoring in employers' homes negatively impacts domestic workers' job satisfaction, and that hongbao gifts from employers significantly promote domestic workers' job satisfaction. The analysis of the causal mechanism based on a structural equation model suggests that video monitoring can increase the discrimination that domestic workers perceive, which in turn reduces their job satisfaction. In particular, we find that domestic workers' perception of discrimination completely mediates the effect of video monitoring on their job satisfaction. However, we also find that hongbao gifts significantly reduce domestic workers' perceptions of discrimination, and thus promote their job satisfaction; that is, the relationship between hongbao gifts and job satisfaction is partially mediated by discrimination. Our study provides a more comprehensive understanding of Chinese employers' labor control strategies and their effects on the job satisfaction of domestic workers.

## Introduction

China has witnessed rapid economic growth and social development since the 1980s, and many women have entered the labor market. Consequently, in most Chinese cities, it is common for middle-class women to have jobs as well as families of their own. This has led to an increasing demand for domestic workers to do housework and to care for children and the elderly. Responding to this demand, migrant women from less developed areas have entered cities, and currently provide a large supply of domestic workers. Owing to the lack of professional norms, the expansion of China's market for domestic work faces two prominent obstacles. First, employers currently lack standardized and effective guidelines for managing domestic workers. Second, domestic workers in China typically have low job stability and satisfaction.

It is important to study domestic workers' job satisfaction because it affects key areas of care needs and regulation of domestic services. In contrast to other service-based occupations, the rate of informal employment among domestic workers is very high (81.2%)—approximately twice the share of informal employment of other employees (39.7%) (1)—which is typical of unstable work. The high instability of domestic work is not only caused by this high rate of informal employment, but also by the ways that employers interact with domestic workers, and specifically their strategies of labor control. From an employer's perspective, the outsourcing of housework and the care of family members to a domestic worker can help alleviate the problems associated with the need to provide private care or the use of public care services, making it possible to achieve work-life balance (2). However, most employers have very limited knowledge about the means for controlling the labor process to maximize the benefits of outsourcing domestic work. Furthermore, labor controls shape the work experiences of domestic workers. Strategies of labor control that are too harsh may reduce the job satisfaction of domestic workers and lead to other negative consequences, such as low job performance (3), low organizational commitment (4), and a high turnover rate (5). These negative effects also represent the feedback of domestic workers on labor controls. Job satisfaction may also affect a domestic worker's motivation and service quality, which in turn affects the health and wellbeing of the recipients of domestic care (6). In general, low work satisfaction is unconducive to the formalization of domestic work and the development of service quality in this specific industry.

The literature suggests that the factors influencing the job satisfaction of domestic workers can be categorized into two areas: the personal characteristics of the domestic worker (e.g., gender, age, marital status, education level, and family background), and the working conditions (e.g., salary, promotion, supervision, and the working relationship) (6–10). Several scholars also emphasize the influence of social support, migration trajectories, work trajectories, and other factors (11–13). However, most of these studies suffer from at least one of the following problems: (1) they lack the perspectives of the immediate employers of domestic workers and their labor control strategies, and instead tend to focus on the employers at the organizational level (e.g., the agents); (2) they tend to be limited in their scope, focusing exclusively on domestic workers that are employed by institutions, or on those who are employed directly by private employers, but never both; and (3) they suffer from methodological limitations; for instance, most studies have been qualitative, and a few quantitative studies have used convenience sampling. Owing to the problems outlined above, limited generalizations can be made about the job satisfaction of domestic workers.

China is presently undergoing rapid change. On the one hand, traditional customs still shape social life; on the other hand, modern technology—especially the Internet—has permeated all sectors of society. In a field survey for the present study, we find that in their interactions with domestic workers, employers in China use modern video-monitoring technology (reflecting a certain degree of distrust by the employer) and also engage in the traditional Chinese custom of “hongbao gifts” (i.e., the presentation of money in a red envelope as a gift; an act indicating intimacy). Accordingly, in this paper we seek to answer the following questions: How do these two contradictory behaviors achieve labor control over domestic workers? Specifically, we examine the impacts of employers' video monitoring (a “tough control”) and hongbao gifts (a “soft control”) on the job satisfaction of domestic workers. As we do not apply a specific sampling frame, we use the Respondent Driven Sampling (RDS) approach to overcome the lack of representation caused by convenience sampling.

## Literature Review and Hypotheses

The concept of “discipline” has a long history in the field of domestic work. Many studies document control strategies used by employers, such as fictive kinship, schedules, and accommodation arrangements (14–19). However, relatively few studies analyze the impacts of labor controls on the job satisfaction of domestic workers or examine the mechanisms by which these impacts unfold.

### Tough Control: Monitoring *via* Video Cameras

Labor control is a core concept in labor process theory. Studies on labor control have mainly focused on organized workplaces (20–23). Although workplace monitoring has been proposed to improve employees' task performance (24), it can also have negative effects on employees (25), such as reducing their job satisfaction, wellbeing, and motivation, increasing their mistrust and other negative emotions, as well as promoting turnover (26, 27). Similarly, studies show that video monitoring can reduce the performances of the monitored persons in simple tasks (28), and create undesirable tension between managers and subordinates (29). Another negative consequence of video monitoring is a reduction in employee job satisfaction (25); this occurs especially when an employee perceives the monitoring as a violation of his or her privacy (30).

Disputes over labor monitoring are rife in domestic work owing to the privacy of the workplace as well as the lack of work standards and privacy protection. Thus, video monitoring may more likely have a negative impact on domestic workers. Domestic workers working in private households—especially those who live with their employers—frequently encounter difficulties in distinguishing between public and private boundaries. Many stay in their employers' houses throughout the duration of their contracts, where they may be made to share a room with the recipient of their care (31). Domestic workers are therefore vulnerable to privacy violations, and some may even lack privacy completely (32). As an “intimate stranger” in the home, the personal life and moral character of a domestic worker is constantly subject to the scrutiny and supervision of their employers. In recent years, the popularization of low-cost monitoring equipment has provided numerous convenient methods for employers to monitor domestic workers (31). The use of telephones for supervision and the discreet installation of “hidden” recorders or cameras in homes to monitor domestic workers have become common practice (33). Such monitoring of domestic workers allows employers to visualize the labor process, though they typically justify their behaviors by claiming that the monitoring is necessary to ensure the safety of individuals in the home (34).

Our survey finds that many employers use cameras because of their low cost and convenience, and their ability to provide real-time monitoring with both images and sound. While employers insist that the cameras are being installed to monitor the recipients of care, domestic workers have shown mixed attitudes toward such installations (i.e., ranging from opposition to support). Only a few qualitative studies document domestic workers' views on video surveillance, such as their dissatisfaction with the installation of cameras (35), which violate their privacy and undermine their trust in their employers (34). However, it is not well known whether such video monitoring affects the work attitudes of domestic workers. We believe that examining the impact of video monitoring on the job satisfaction of domestic workers provides a more indirect indication of domestic workers' genuine attitudes toward video monitoring.

Accordingly, we propose the following hypothesis.

H1: The installation of video cameras in the home of the employer significantly lowers the job satisfaction of the domestic worker.

### Soft Control: Hongbao Gifts

In traditional Chinese culture, “hongbao” are gifts in the form of cash in envelopes, which are presented for the purpose of maintaining relationships (36). Hongbao are generally used to consolidate social relationships. For instance, they can be used as a means for strengthening one's connection with relatives and friends, or to confirm one' s personal relationships. In China' s transformation from a traditional society of “acquaintances” to a modern society of “strangers,” the custom of giving hongbao has extended beyond the scope of one's relatives and friends; it has penetrated the industrial and commercial environment, where it commonly modulates the relationship between employer and employee.

Monetary rewards have shown to improve employee job satisfaction in many different sectors of work (37, 38), and in particular, financial returns have been shown to enhance employee job satisfaction in emotional labor (39). In China, employers often give hongbao to their employees at the beginning and end of the year, as well as during major festivals and celebrations. Such hongbao-giving not only serves the purpose of providing the employee with an economic incentive, but also—and more importantly—enhances the emotional connection between employer and employee, thereby increasing the likelihood that the employee will adhere to the employer's subsequent instructions. For instance, one study finds that presenting cash in the form of a hongbao rather than as ordinary cash, leads to a greater improvement in employees' overall productivity, willingness to participate, and work quality (40).

The custom of giving hongbao can likewise be beneficial to the construction of an informal relationship between employers and domestic workers. Some domestic workers are willing to establish an intimate relationship with their employers, and they may proactively pursue this. They may regard the development of intimate relationships as a sign of their employers' respect (41–43). From these intimate relationships, domestic workers may also derive emotional recognition, which they cannot obtain simply from their employment contracts (44). Research shows that acknowledgment indicates that the recipient is worthy of trust, respect, and appreciation (45). Additionally, employees' job satisfaction tends to increase when they perceive respect from their employers or managers (46, 47). We expect that similar direct psychological rewards induced by hongbao gifts will stimulate the transformation of relationships based on paid work to emotional relationships. In our opinion, as the medium of personal relationships, hongbao is the means used by employers to control the labor of domestic workers. Domestic workers who accept hongbao are more likely to be controlled by their employers. On the one hand, hongbao may have a positive impact on the labor process and results of domestic workers; on the other hand, it could consolidate unstable employment relationships of domestic service. Therefore, we predict that hongbao gifts will positively affect the job satisfaction of domestic workers.

H2: An employer's hongbao gift has a significant positive effect on the job satisfaction of the domestic worker.

### Mediating Variable: Perceived Discrimination

Domestic workers are a stigmatized group (48, 49). Often, this discrimination relates to the gender or race of the domestic worker, or the enslavement traditions and class structure in the country of work (17, 50–52). The position of domestic workers on the fringe of the labor market also reflects the exclusive labor policies in some countries or districts (53, 54). These factors (gender, race, traditional class, and labor policies) also cause domestic workers to have low professional statuses in society, and make them a group vulnerable to discrimination. Furthermore, domestic workers are vulnerable to discrimination in the workplace. Interpersonal discrimination, a component of workplace discrimination, includes negative verbal and nonverbal behaviors that occur in daily social interactions in the workplace (55). In comparison with other employees in the service industry, domestic workers spend greater amounts of time with their employers, interact more frequently, and are more likely to be the subjects of interpersonal discrimination. For instance, employers who play the roles of benevolent mothers believe that their domestic workers are weaker than themselves, and therefore need to be protected. Such employers confirm their class status and superiority in daily dealings (14, 56, 57). In contrast, their domestic workers may perceive discrimination. One meta-study shows that perceived discrimination is significantly related to job satisfaction (55).

Numerous laws make clear provisions on discrimination in the workplace to prevent workers from being treated unfairly. Nonetheless, workplace discrimination continues to be manifested in inconspicuous behaviors and attitudes, placing the recipient of such discrimination at a considerable disadvantage. As suggested by Jones et al. (58) while public discrimination is resisted by society, subtle discrimination is difficult to define and eliminate. Because employers in the private sector possess considerable power to decide and interpret their actions, it is difficult to judge their true intentions owing to the unclear boundaries between that which is “public” and that which is “private.” It is also difficult to determine whether certain behaviors of employers toward domestic workers are shaped by prejudices. Therefore, domestic workers' perceptions may influence their understanding and interpretation of the behaviors of their employers. Cameras capture the real-time labor process of domestic workers. Such video monitoring may cause domestic workers to feel as though they are being discriminated against by their employers, thereby reducing their job satisfaction. For instance, a recent study shows that electronic monitoring affects employee satisfaction by changing employee cognition (59). Likewise, we expect that video monitoring will affect the job satisfaction of domestic workers (i.e., through their respective attitudes to video monitoring). In contrast, we expect that the potential signs of gratitude and the recognition that employers convey through giving hongbao will reduce the discrimination perceived by domestic workers, thereby improving their job satisfaction.

Accordingly, the following hypotheses are proposed.

H3: Perceived discrimination mediates the effect of video monitoring on job satisfaction.
H4: Perceived discrimination mediates the effect of hongbao gifts on job satisfaction.

Material factors, such as an adequate compensation or a safe and comfortable physical environment, are key to ensuring the job satisfaction of domestic workers (60). If video monitoring—which is a “tough” means for controlling the labor of domestic workers—destroys the safety and comfort of the working environment; in contrast, hongbao gifts—a kind of “soft” control strategy—are a form of economic and psychological compensation that employers present to their domestic workers. We expect that employers' labor control over domestic workers can be strengthened *via* the use of either “tough” or “soft” strategies.

**Figure 1:**
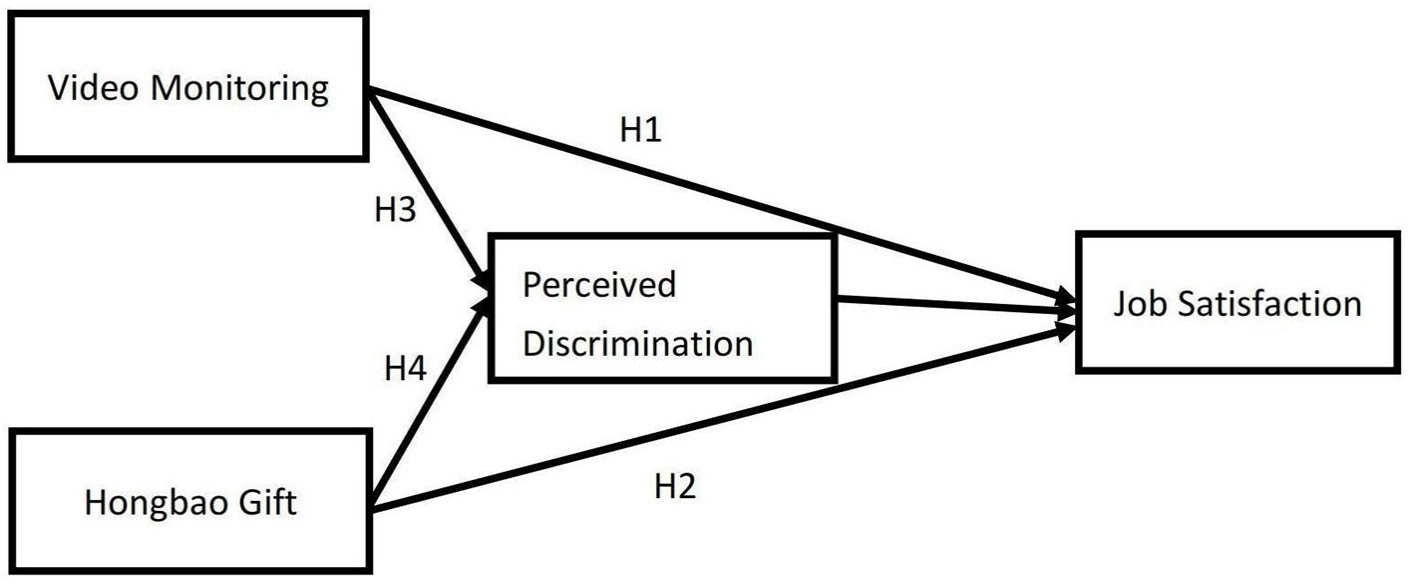
Impact mechanism of the effects of video monitoring and hongbao gifts on the job satisfaction of domestic workers.

Based on the above discussion, we propose a research framework (Figure 1).

## Methods

### Data and Respondents

The research data originated from a questionnaire-based survey on domestic work, which was conducted in four cities in China (Nanjing, Wuxi, Guangzhou, and Foshan) from June to August 2019. The surveys were conducted by the “Research on the Employment of Domestic Workers in the Internet Era” research group in the School of Social and Behavioral Sciences, Nanjing University. Domestic workers are usually employed in an informal capacity and many are not officially registered. For this reason, a sampling frame of domestic workers was difficult to obtain. We used the RDS approach in the survey to address this problem, “because standard probability sampling methods produce low response rates and responses that lack candor” (61). RDS is a network sampling technique that is typically employed to sample hidden populations that lack a sampling frame (62). The results of the RDS indicated that the dataset was representative. We explained the purpose of the research and obtained the informed consent from each participant prior to the survey.

The respondents were domestic workers who had been serving in their employers' houses for extended periods of time (i.e., more than or equal to 3 months). To reduce potential sources of bias in our analysis, our surveys excluded domestic workers who had worked for their employers for short periods of time (i.e., <3 months), or who were paid by the hour, as such domestic workers usually served multiple employers during the same period. We also excluded eight domestic workers who were male, because these samples insufficiently represented the population of male domestic workers. Our final sample consisted of 699 domestic workers who ranged between the ages of 28 and 70 years (M = 50.84, SD = 6.62). Approximately 88.4% of the respondents were married, and more than 70% of them originated from rural areas. The majority of respondents (86%) had a junior high school or lower educational level.

### Measurements

#### Dependent Variable

##### Job Satisfaction

The “overall job satisfaction scale” developed by Tsui et al. (63) is among the most widely used by researchers^1^. The scale includes six items that measure an individual's satisfaction with his or her work performance, organizational superiors, relationships with co-workers or peers, income, and promotion opportunities, as well as the overall job satisfaction. Domestic workers typically do not maintain any relationships with their co-workers (as these usually do not exist in the homes of their employers) or their superiors, and job promotions are extremely rare. Accordingly, we modified the scale by eliminating the item measuring the individual's satisfaction with a co-worker-relationship, and replacing the items measuring the individual's satisfaction with the superior-relationship and promotion opportunities with items that measured the individual's satisfaction with the employee–employer relationship and the occupational status. Thus, our modified scale included a total of five items; it measured a domestic worker's satisfaction with her job performance, income, employee–employer relationship and occupational status, as well as her overall job satisfaction. Respondents' answers to each item were rated on a five-point Likert scale that ranged from a value of 1 (“very dissatisfied”) to a value of 5 (“very satisfied”). The Cronbach's alpha for the scale was 0.75, and the KMO was 0.763 (*p* < 0.001); this indicated that the scale had satisfactory reliability.

#### Independent Variables

##### Video Monitoring

The participants were asked “Does your employer have a video camera installed in the home at present?” The response items were “yes,” “no,” and “unsure.” Among the domestic workers surveyed, 25% of the participants clearly stated that their employers had video cameras installed in their homes, 66.48% said that their employers had not installed such devices, and 8.06% said that they were unsure as to whether any such devices had been installed. We combined the data for the “no” and “unsure” responses and coded them with a value of 0; we coded the “yes” responses with a value of 1.

##### Hongbao Gift

The participants were asked “Did you receive a hongbao from your employer in the previous year?” The response items were “yes” and “no.” If a participant answered “yes,” she was subsequently asked to state the monetary value of the hongbao she received. Among the domestic workers surveyed, 62% reported receiving hongbao from their employers. The values of hongbao received ranged from 5 to 10,000 yuan (M = 1,084.18, SD = 1,480.04). We used hongbao value (in yuan) as an independent variable, coded participants who had not received hongbao with a value of 0, and performed logarithmic processing (M = 3.92, SD = 3.2).

#### Mediating Variable

##### Perceived Discrimination

We measured the discrimination the domestic workers perceived by asking the question “Have you ever felt discriminated against because of your status as a domestic worker?” For the response items, we used a five-point scale, which ranged from a value of 1 (“never”) to a value of 5 (“always”). Among the domestic workers surveyed, 61.39% reported “never” feeling discriminated against; while 21.92%, 10.33%, and 6.36% of the domestic workers reported feeling discriminated against “occasionally,” “sometimes,” and “often” or “always,” respectively. We treated Perceived Discrimination as a binary variable, that is, coding a response of “never” with a value of 0, and all other responses with a value 1.

#### Control Variables

Our model controlled for any variables that corresponded to participants' sociodemographic characteristics and work statuses (Table 1). The social demographic variables included the age, years of education, household registration (rural = 0, urban = 1), and marital status (unmarried = 0, married = 1) of each participant. The variables corresponding to participants' work statuses included the number of years for which they had worked as domestic workers (i.e., a continuous variable, in years), the type of work they performed (housework = 0, child care = 1, elderly care = 2), whether they were living in their employer's house (no = 0, yes = 1), whether they had signed a contract with their employer (no = 0, yes = 1), and their monthly salary (logarithmic).

**Table 1:** Descriptive statistics for the control variables (*N* = 699).

**Variable**	**Mean**	**Std. dev**.	**Min**	**Max**
Age	50.84	6.63	28	70
Years of education	8.17	2.96	0	16
Household registration (urban = 1)	0.26	0.44	0	1
Marital status (married = 1)	0.88	0.32	0	1
Years of work as a domestic worker	7.77	6.53	0	38
**Type of work**
Housework = 0	0.17	0.38	0	1
Child care = 1	0.50	0.50	0	1
Elderly care = 2	0.33	0.47	0	1
Live-in or live-out domestic worker (live-in = 1)	0.74	0.44	0	1
Employment contract (yes = 1)	0.56	0.50	0	1
Monthly salary (logarithmic)	8.46	0.41	6.91	9.79

### Data Analysis

We used a structural equation model (SEM) in the software Stata16 to estimate the relationships between Video Monitoring, Hongbao Gifts, Perceived Discrimination, and Job Satisfaction. As the mediating variable Perceived Discrimination was a binary variable, we used the “gsem” command in Stata16 to fit this binary variable to estimate the model parameters. However, the “gsem” command could neither directly report the goodness-of-fit for the model, nor calculate the direct, indirect, or total effects. Therefore, in a second analysis, we included Perceived Discrimination as a continuous variable. We used the “SEM” command to estimate the model parameters in a linear probability model, and calculated the model's goodness-of-fit, and the direct, indirect, and total effects. Finally, we used the bootstrap method to estimate the direct, indirect, and total effects of the independent variables.

## Results

The results of the SEM estimated using the maximum-likelihood method are presented in Table 2. Perceived Discrimination was estimated using a logit model in Model 1 and using an OLS model in Model 2. Based on the results of the goodness-of-fit indices, both the SRMR and RMSEA of Model 2 were < 0.05, indicating a good fit. However, the values of log-likelihood, AIC and BIC in Model 1 were smaller than those of Model 2, indicating that a better fit was achieved using the logit model.

**Table 2:** Results of SEM analysis for domestic workers' job satisfaction.

		**Model 1**	**Model 2**
**A. Measurement model**			
Job satisfaction	Job performance		
	_cons	2.761***(0.545)	2.761***(0.545)
	Income	1.269***(0.141)	1.269***(0.141)
	_cons	1.751*(0.692)	1.751*(0.692)
	Employee–employer relationship	1.125***(0.113)	1.125***(0.113)
	_cons	2.704***(0.601)	2.704***(0.600)
	Occupational status	1.379***(0.143)	1.379***(0.143)
	_cons	1.911**(0.733)	1.911**(0.733)
	Overall job satisfaction	1.002***(0.080)	1.002***(0.080)
	_cons	2.957***(0.540)	2.957***(0.540)
**B. Structural model**			
**—>** **Perceived discrimination**		(Logit model)	(Linear probability model)
Video monitoring (no or unsure = 0)		0.320**(0.121)	0.076**(0.028)
Hongbao gift (logarithmic)		−0.074**(0.024)	−0.017**(0.006)
_cons		−0.317*(0.132)	0.422***(0.031)
**—>Job satisfaction**			
Perceived discrimination (never = 0)		−0.258***(0.042)	−0.258***(0.042)
Video monitoring (no or unsure = 0)		−0.045(0.028)	−0.045(0.028)
Hongbao gift (logarithmic)		0.028***(0.006)	0.028***(0.006)
Age		0.007*(0.003)	0.007*(0.003)
Years of education		−0.006(0.007)	−0.006(0.007)
Household registration (urban = 1)		0.028(0.045)	0.028(0.045)
Marital status (married = 1)		0.074(0.056)	0.074(0.056)
Years of work as a domestic worker		0.003(0.003)	0.003(0.003)
**Type of work**			
Housework = 0		−0.057(0.055)	−0.057(0.055)
Child care = 1		−0.172**(0.058)	−0.172**(0.058)
Elderly care = 2		0.031(0.044)	0.031(0.044)
Live-in or live-out domestic worker (live-in = 1)		−0.024(0.036)	−0.024(0.036)
Employment contract (yes = 1)		0.163**(0.058)	0.163**(0.058)
**C. Goodness-of-fit indices**			
		Log likelihood = 4347.209; AIC = 8760.418, BIC = 8910.932	Log likelihood = −14855.129; AIC = 29778.259, BIC = 29933.334; SRMR = 0.038, RMSEA = 0.044; R-squared = 0.158
*N*		699	699

* *p < 0.05*,

** *p < 0.01*,

*** *p < 0.001*.

In both Models 1 and 2, the factor loadings of the four latent variables were all significant at the 5% level, indicating that the measurement model was effectively accepted. Models 1 and 2 both showed that the installation of video monitoring in employers' homes had no significant impact on domestic workers' job satisfaction. However, Video Monitoring showed a significant positive effect on perceived discrimination (*p* = 0.01). That is, domestic workers who were subject to video monitoring were more likely to perceive themselves as being discriminated against. This result indicated that video monitoring did not directly impact the job satisfaction of domestic workers, but had an indirect negative impact on their job satisfaction through the mediating effect of perceived discrimination.

The estimated coefficient for Hongbao Gift indicated that it had a positive effect on the job satisfaction of domestic workers (*p* = 0.001). Specifically, for every 1% increase in Hongbao Gift, Job Satisfaction increased by 2.8%. Hongbao Gift also had a significant negative impact on Perceived Discrimination (*p* = 0.01). That is, a domestic worker who received a hongbao gift of greater value from her employer was less likely to feel discriminated against. Furthermore, Perceived Discrimination was found to have a negative impact on the job satisfaction of domestic workers (*p* = 0.001). Domestic workers who had perceived discrimination experienced 25.8% lower job satisfaction than others who had never perceived discrimination. The results showed that in addition to directly promoting job satisfaction, hongbao gifts could also promote job satisfaction indirectly by reducing perceived discrimination.

To obtain the total (i.e., net) effect of Hongbao Gift, Video Monitoring, and Perceived Discrimination on Job Satisfaction, we reported their standardized coefficients based on Model 2 (as shown in Table 3).

**Table 3:** Standardized direct effects, indirect effects, and total effects of hongbao gifts, video monitoring, and perceived discrimination on the job satisfaction of domestic workers.

	**Std. Coef**.	**Std. Err**.	**Z**	***P***
**Standardized direct effects**				
Video monitoring —> Job satisfaction	−0.067	0.028	−1.62	0.105
Hongbao gift —> Job satisfaction	0.209	0.006	4.65	0.000
Video monitoring —> Perceived discrimination	0.100	0.028	2.690	0.007
Hongbao gift —> Perceived discrimination	−0.114	0.006	−3.080	0.002
Perceived discrimination —> Job satisfaction	−0.294	0.042	−6.17	0.000
**Standardized indirect effects**				
Video monitoring —> Job satisfaction	−0.029	0.008	−2.470	0.014
Hongbao gift —> Job satisfaction	0.034	0.002	2.750	0.006
**Standardized total effects**				
Video monitoring —> Job satisfaction	−0.097	0.029	−2.250	0.025
Hongbao gift —> Job satisfaction	0.243	0.006	5.180	0.000
Video monitoring —> Perceived discrimination	0.100	0.028	2.690	0.007
Hongbao gift —> Perceived discrimination	−0.114	0.006	−3.080	0.002
Perceived discrimination —> Job satisfaction	−0.294	0.042	−6.170	0.000

With reference to the standardized coefficients in Table 3, we found that the direct effect of Video Monitoring on Job Satisfaction was not significant. Instead, we found that its indirect effect and total effect were both significantly negative at the 0.05 level (−0.029, *p* = 0.014; and −0.097, *p* = 0.025, respectively). This indicated that Video Monitoring reduced Job Satisfaction through Perceived Discrimination, in a complete mediation effect. Hongbao Gift had both a positive direct effect (0.209, *p* = 0.000) and a positive indirect effect (0.034, *p* = 0.006) on Job Satisfaction. The sum of the two variables—Video Monitoring and Hongbao Gift—yielded the total effect on Job Satisfaction (0.243, *p* = 0.000). These results show that the role of Hongbao Gift on Job Satisfaction is part of the mediating effect.

## Discussion

There is an urgent need to promote the job satisfaction of domestic workers in China due to the increasing demands for housework and care, as well as the low professional standards of domestic work and the high mobility of domestic workers in the country. In this study, we investigate how domestic workers' job satisfaction is influenced by two labor control strategies used by Chinese employers: video monitoring, which has rapidly become popular in the private sector, and hongbao gifts, which have the function of embedding personal relationships within formal relationships. The former strategy of labor control uses cameras to extend panopticism to the private space; the latter is a means for employers to exploit “strategic intimacy” (64) and control domestic workers to ensure their service quality, or to seek unpaid labor (65). In addition, we find that domestic workers' perceptions of employer behaviors, specifically whether domestic workers feel discriminated against, play an important role in the impact of labor control on job satisfaction.

Applying SEM to data from surveys of domestic workers in four cities in China, we examine how the two labor control strategies—the installation of video cameras in the home for monitoring purposes and the giving of hongbao to domestic workers—affect the job satisfaction of domestic workers. The results verify our hypotheses that video monitoring and hongbao gifts significantly impact domestic workers' job satisfaction. Specifically, we find that video monitoring negatively affects domestic workers' job satisfaction and this relationship is completely mediated by perceived discrimination. In contrast, hongbao gifts not only directly and positively affect domestic workers' job satisfaction but also have an indirect positive effect by reducing perceived discrimination.

The installation of video cameras in the home completely exposes the labor process of a domestic worker to an employer. Decisions on whether and where to install such cameras—and whether to inform and obtain the consent of domestic workers—lie with individual employers. The uncertainty about the number and locations of cameras, as well as the unknown identities of the observers and the duration of monitoring make video monitoring more invasive than other forms of monitoring (34). The professional and private lives of domestic workers overlap considerably in both time and space, and it is difficult for these individuals to find “safe spaces” where they can escape the camera's uninterrupted gaze. The installation of video cameras in homes therefore causes domestic workers to worry about privacy violations and to believe that they are not trusted by their employers.

It is worth noting that the domestic workers' perceived discrimination completely mediates the effect of video monitoring on their job satisfaction. This suggests that the video camera stimulates a domestic worker's perception of discrimination, and negatively affects her job satisfaction through this mechanism. Prior to entering employment in a private household, some domestic workers may already be aware that their occupation is discriminated against, as domestic work is sometimes treated as a “dirty” or “inferior” form of work. This perception may change in response to the behavior of the employers. More than half of the domestic workers surveyed indicated that they found it acceptable for their employers to install video cameras in their homes. However, this does not eliminate the negative effects of such monitoring. Domestic workers' attitudes toward cameras reflect their recognition of their own identity as “others” and their lack of “voice” in the private family. This represents a helpless and passive acceptance of the asymmetrical power relations. Some domestic workers even see video monitoring as a means to prove their integrity and to advertise their service quality. For instance, one worker caring for newborns and their mothers squared her shoulders and stated that she “very much supported (her employer installing a video camera in the home)” because “a clean hand wants no washing,” and thus video monitoring could help her employers address any suspicions they might have had concerning her integrity.

Hongbao gifts are not only means for employers to encourage domestic workers to work hard and work more, but are also concrete representations of personal relationships in the Chinese context. The efficacy of hongbao gifts in domestic services can operate through the following channels. First, hongbao have economic value. In contrast to non-monetary gifts with less tangible economic values, the economic returns or compensation represented by a hongbao is clear. Second, hongbao act as incentives, conveying employers' recognition and endorsement of the work quality or personality of domestic workers, and their expectations for subsequent high-quality services, which may motivate the domestic workers. Third, hongbao act to embed emotional connections within a contractual relationship based on market logic. In traditional Chinese culture, the giving and receiving of hongbao takes place primarily during the Spring Festival, and is an intergenerational gift. In this context, hongbao represent the blessing of the giver on the recipient, and serves to reinforce the bonds with one's family, relatives, and friends. In contrast, the timing of employers' hongbao are not fixed; they may be presented on holidays or on the birthdays of domestic workers. In this context, hongbao represent employers' blessings and gratitude to domestic workers, strengthening the emotional connections between the two parties. This emotional connection, as well as the variable timing at which hongbao are presented, blurs the perception of unequal status in the employer–employee relationship, even providing domestic workers with some psychological return and increasing their loyalty. Finally, hongbao can function as a medium for personal relationships and thus reduce the mobility of domestic workers and stabilize their employment. In contrast to foreign domestic workers who operate in conditions of “legal servitude” (66), domestic workers encounter low costs in changing jobs in mainland China, allowing them relatively high mobility. The service quality of domestic workers in the current market varies considerably, and it is difficult to find “good” domestic workers. Labor controls such as video monitoring that potentially risk violating human rights may become reasons for domestic workers to actively terminate their contracts. In contrast, hongbao gifts are taken to represent the favor and face of the employers. In Chinese culture, gifts help to maintain the informal relationship between the two interacting parties. A rejection of a gift is interpreted as the rejection of the giver's kindness; such actions risk damaging the face of the giver and destroying the relationship. Therefore, employers use hongbao gifts and foster personal relationships to ease the inherent tension in domestic services. These labor controls can act as soft constraints on domestic workers' abilities or willingness to leave their jobs, and therefore reduce their mobility. In addition to increasing the income and internal motivation of domestic workers, which increase directly with their job satisfaction, the psychological return of respect and recognition brought about by hongbao gifts reduces domestic workers' perceptions of discrimination, and indirectly promotes their job satisfaction.

In summary, our research demonstrates the distinct effects of tough labor controls and soft labor controls on the job satisfaction of domestic workers and verifies the mediating effect of domestic workers' cognition (i.e., perceived discrimination). In addition, our analysis of domestic workers' perceptions of discrimination aligns with the conclusions of previous studies, which show that discrimination at work negatively impacts an individual's job satisfaction (4, 67–69). The two strategies continue to strengthen the labor control of domestic workers. The camera has introduced a novel, accessible means of labor control to employers—precisely at a time when few are able to supervise the work of domestic workers because of their own work commitments. The positive signals conveyed by hongbao gifts substantiate employers' gratitude in exchange for the work of domestic workers. However, hongbao are nonetheless a disguised form of labor control, designed to offset or alleviate the negative perceptions and work attitudes brought about by video monitoring.

Our study makes three main contributions. First, we incorporate video monitoring, hongbao gifts, and perceived discrimination into an analytical framework for understanding the labor processes of domestic workers; we discuss employers' tough- and soft control strategies and the mechanisms through which domestic workers' cognition impacts their job satisfaction. Second, our study highlights how domestic workers' identities are shaped by modernity and society and emphasizes the influence of employers' behaviors on the attitudes of domestic workers to their jobs. Third, our quantitative analysis using the representative samples obtained by RDS sampling complements qualitative research on domestic work.

To be clear, we are not calling for the prohibition of video monitoring or recommending that all employers present hongbao gifts to their domestic employees. Instead, by examining the impacts of two labor control strategies on the job satisfaction of domestic workers, we wish to draw attention to the subjective experiences of domestic workers. As one important limitation, the present research does not account for differences in the types of employment relationships, which have shown to play an important role in shaping employers' choices and motivations for labor control strategies, as well as the responses of domestic workers (64). However, owing to limitations in the data, we do not consider different types of employment relationships and their complex interactions; neither do we account for the emotional experience between employers and employees in this study. In addition, the symbolic meanings of hongbao gifts vary across different regional cultures in China. Such regional and cultural differences may influence domestic workers' perceptions of hongbao, and thus altering hongbao effects on job satisfaction and job performance. Future studies may therefore expand on the scope of the present research by exploring how the effects of different types of employment relationships and their interactions, the emotional experiences of employers and employees, and regional culture influence labor controls of domestic workers and their attitudes toward these controls.

## Data Availability Statement

The raw data supporting the conclusions of this article will be provided by the corresponding author upon request.

## Author Contributions

LL contributed to the conceptualization and revision. AY and SF contributed to methodology and writing the manuscript. CF conducted the data analyses and critically revised the manuscript. MJ reviewed and edited the manuscript. All authors reviewed and approved the submitted version.

## Funding

This study was funded by the Philosophy and Social Science Foundation of China (Grant-No. 18ASH007).

## Conflict of Interest

The authors declare that the research was conducted in the absence of any commercial or financial relationships that could be construed as a potential conflict of interest.

## Publisher's Note

All claims expressed in this article are solely those of the authors and do not necessarily represent those of their affiliated organizations, or those of the publisher, the editors and the reviewers. Any product that may be evaluated in this article, or claim that may be made by its manufacturer, is not guaranteed or endorsed by the publisher.

## Footnotes

^1^Given the differences between domestic work and other occupations, we did not choose a complicated job satisfaction scale.
